# Rehabilitation access and effectiveness for persons with back pain: the protocol of a cohort study (REHAB-BP, DRKS00011554)

**DOI:** 10.1186/s12889-017-4588-x

**Published:** 2017-07-14

**Authors:** Matthias Bethge, Kerstin Mattukat, David Fauser, Wilfried Mau

**Affiliations:** 1 Institute for Social Medicine and Epidemiology, University of Lübeck, Ratzeburger Allee 160, 23562 Lübeck, Germany; 2 Institute for Rehabilitation Medicine, Martin Luther University Halle-Wittenberg, Magdeburger Str. 8, 06112 Halle, Germany

**Keywords:** Medical rehabilitation, Back pain, Access barriers, Effectiveness, Propensity score matching

## Abstract

**Background:**

Back pain is one of the most common chronic diseases in Germany and has a major impact on work ability and social participation. The German Pension Insurance (GPI) is the main provider of medical rehabilitation to improve work ability and prevent disability pensions in Germany. However, over half of the persons granted a disability pension have never used a medical rehabilitation service. Furthermore, evidence on the effects of medical rehabilitation in Germany is inconclusive. Consequently, this study has two aims: first, to determine barriers to using rehabilitation services, and second, to examine the effectiveness of medical rehabilitation in German residents with chronic back pain.

**Methods:**

In 2017 a postal questionnaire will be sent to 45,000 persons aged 45 to 59 years whose pension insurance contributions are managed by the GPI North or the GPI Central Germany. In 2019 respondents who report back pain in the first survey (*n* = 5760 expected) will be sent a second questionnaire. Individuals will be eligible for the first survey if they are employed, have neither used nor applied for a rehabilitation programme during the last 4 years and neither received nor applied for a disability pension. The sample will be drawn randomly from the registers of the GPI North (*n* = 22,500) and the GPI Central Germany (*n* = 22,500) and stratified by sex and duration of sickness absence benefits. Barriers to rehabilitation services will be related to socio-demographic and social characteristics, pain and attitudes to pain, health and health behaviour, healthcare utilisation, experiences and cognitions about rehabilitation services and job conditions. Propensity score matched analyses will be used to examine the effectiveness of rehabilitation services. Data on use of medical rehabilitation will be extracted from administrative records. The primary outcome is pain disability. Secondary outcomes are pain intensity and days of disability, pain self-efficacy, fear avoidance beliefs, self-rated health, depression, healthcare utilisation, self-rated work ability and subjective prognosis of employability, sickness absence benefits, and disability pensions.

**Discussion:**

This study identifies barriers to use of rehabilitation services and determines the effectiveness of medical rehabilitation for patients with chronic back pain.

**Trial registration:**

German Clinical Trials Register (DRKS00011554, January 26, 2017).

**Electronic supplementary material:**

The online version of this article (doi:10.1186/s12889-017-4588-x) contains supplementary material, which is available to authorized users.

## Background

With 12-month prevalence rates of more than 75% [1], back pain is one of the main health problems in German adults [2]. National health surveys indicate that 25% of women and 17% of men reported experiencing chronic back pain (CBP), i.e. back pain almost every day for at least 3 months, during the last 12 months [3]. Back pain is more prevalent in women, in older people and in people with less education [1, 3]. Use of health care services, sickness absence, and disability pension payments mean that illness has a high cost [2].

The World Health Organisation (WHO) deemed rehabilitation essential to enabling people with disabilities or chronic diseases to participate in work and society, but also identified unmet needs [4]. In Germany, age standardised utilisation of medical rehabilitation for musculoskeletal disorders decreased over the 14-year period between 2001 and 2015 from 11.9 per 1000 to 8.7 per 1000 in men and from 10.6 per 1000 to 7.7 per 1000 in women. Moreover, the proportion of disability pensioners with musculoskeletal disorders who did not undergo medical rehabilitation before their disability pension was granted increased over the 5-year period between 2007 and 2012 (males: 47.5 to 48.3%; females: 41.0 to 45.0%) [5, 6].

Previous international research studies have reported various barriers to using rehabilitation services, i.e. factors that prevent persons from using rehabilitation services despite an obvious need for them. For example, a systematic review [7] identified a range of financial, structural, personal and attitudinal determinants of access to rehabilitation services in the United States, but it is questionable whether these findings would transfer to the German context. In Germany, rehabilitation for working-age people is provided mainly by the German Pension Insurance (GPI). The GPI is a compulsory pension insurance scheme. Workers contribute to a pension scheme which is administered by the GPI. If workers reach the age of 65 years (gradually increasing to 67 years by 2031), the GPI pays a monthly pension based on their pension contributions. People who have a permanent disability that prevents them from working receive a disability pension until they become eligible for an old age pension. The GPI provides rehabilitation services on the principle ‘rehabilitation before pension’, i.e. they aim at avoiding paying disability pensions. The GPI offers about one million rehabilitation programmes per annum. Usually the prerequisite for access to rehabilitation is a claim by the person in question. The claim will be appraised by the GPI to determine the need for rehabilitation. Only post-acute rehabilitation (about one third of all rehabilitation measures) makes a simplified procedure for gaining access to medical rehabilitation possible.

During the last two decades, a number of studies have attempted to address the problem of unmet rehabilitation needs in Germany, either by asking people eligible for rehabilitation services or important stakeholders about barriers to using them [8–13] or by comparing users and non-users to identify determinants of rehabilitation utilisation [13–19]. According to these studies, the main predictor of rehabilitation utilisation appears to be impaired health, manifested as poor subjective health [8, 11, 14–17], functional impairment or impairment in activities of everyday life [11, 19], chronic conditions [11, 19], poor work ability [11, 18], long sickness absence [8, 11, 14, 19], and greater use of medical care services [11, 14]. Though these findings indicate that use of medical rehabilitation services is linked to substantial health problems affecting work ability, they do not give insight into the barriers to using rehabilitation services. Other studies have found that subjective rehabilitation need, rehabilitation intention and plans for utilising rehabilitation services predicted use of services [17]. These variables may be proxy measures for the level of information about rehabilitation services and thus lack of awareness may represent a barrier to use of services. This suggestion is supported by the finding that the probability that an individual would request rehabilitation services was increased by a higher self-efficacy, i.e. the belief of being able to apply for a medical rehabilitation [17]. Further cross-sectional findings also indicate the relevance of self-rated job insecurity [8, 9, 11, 13], the recommendation of the attending physician [8, 10–12, 17, 20] and perceived family support [10–12, 17, 20]. Positive attitudes toward rehabilitation services were more salient in former rehabilitants or persons who planned to participate in a rehabilitation programme in the near future [8, 11, 19, 20]. Previous research studies have produced inconsistent findings on the roles of sex, age, and socio-economic background in the use of rehabilitation services [8, 11, 13–15, 20]. In the case of people with CBP intention to use and actual use of rehabilitation services may be influenced by job situation, work conditions, conflict between work and family roles and coping strategies, but so far these potential barriers have not been investigated through longitudinal research. Our first research goal is therefore to analyse a comprehensive set of barriers to accessing rehabilitation via a prospective design, using a sample large enough to provide a clear picture of the determinants of use of medical rehabilitation services.

The second research goal relates to the effectiveness of rehabilitation services. Attending physicians, who are important stakeholders when requesting medical rehabilitation, should base their advice about medical rehabilitation on the best current evidence [21]. Although international research has shown that multimodal medical rehabilitation has a beneficial impact on various outcomes (work ability, pain, quality of life, participation) [22–27], the setting and context of these studies were different from the 3-week inpatient rehabilitation programmes that are usual in Germany and so a separate evidence base is needed to demonstrate the effectiveness of German rehabilitation programmes. However, evidence on the health-related effects of German medical rehabilitation programmes for patients with CBP is, at best, contradictory [28, 29]. A previous small, single-centre randomised controlled trial tested the efficacy of medical rehabilitation in patients with CBP using a waiting list control group. Jäckel et al. [30] found short-term effects (4-week follow-up) on pain, anxiety and depression. The design did not enable testing of long-term effects. A more recent randomised controlled trial by Hüppe et al. [31] used a smart approach in which patients suffering from chronic musculoskeletal disorders who had impairments indicative of a need for rehabilitation had been actively supported to request for rehabilitation services. Within 6 months of study entry, 69% of the intervention participants, but only 20% of the controls, participated in a 3-week inpatient rehabilitation programme, but there were no group differences in any of the primary and secondary outcomes at the 6- and 12-month follow-ups. It should be noted, however, that the effects of rehabilitation were tested in a population that only opted to pursue rehabilitation after receiving additional support and counselling designed to encourage them to do so. Lower grade evidence for the effectiveness of rehabilitation is available from case series (observational studies without controls). These studies reveal minor to moderate improvements in several outcomes [32, 33]. However, the randomised controlled trial by Hüppe et al. [31] indicated that comparable patients who do not use rehabilitation services might show similar improvements. Whilst these findings might justify restricting access to medical rehabilitation, remarkable efforts have been made to improve rehabilitation services over the last several years. These include the development of evidence-based therapy standards designed to improve the quality of multi-professional treatment and the provision of active therapies [34], implementation of modern patient education interventions [35, 36], the development of work-related medical rehabilitation [37–40], and more emphasis on aftercare and follow-up sessions to maintain behaviour and attitude changes [41, 42]. Randomised controlled trials and controlled clinical trials have shown that these modifications do improve outcomes compared to conventional medical rehabilitation.

According to the demands of evidence-based medicine, the lack of evidence may itself be a major barrier to the utilisation of rehabilitation services. It follows that to justify increased utilisation of rehabilitation services in the future we need high-quality investigations into the effectiveness of rehabilitation services. From a health service research perspective there needs to be a focus on rehabilitation under real-life conditions and its long-term effects through comparisons with patients who do not receive rehabilitation services. Large-scale, randomised controlled trials with long-term follow-up periods are not feasible due to the legal foundations of rehabilitation, so appraisals of the outcomes of medical rehabilitation must be based on data from cohort studies. To our knowledge, this study is the first attempt to investigate the effects of German medical rehabilitation services for persons with CBP, under routine conditions and via a large cohort study using propensity score matching.

The study protocol has been prepared according to the Standard Protocol Items: Recommendations for Interventional Trials (SPIRIT) checklist [43].

## Methods

### Study design

The study is designed as a cohort study. Persons with back pain will be identified via a preliminary survey and then followed up. Back pain patients who complete a medical rehabilitation will be compared with back pain patients who do not participate in a medical rehabilitation programme. Propensity score matching will be used to match controls to treated patients and to estimate unbiased effects of medical rehabilitation.

Baseline data will be assessed to identify persons with back pain and to analyse barriers to using rehabilitation services. Follow-up data will be assessed at the beginning of the third year of the study to determine the effectiveness of medical rehabilitation. Administrative data from the GPI registers will also be used. Given the observational design no-one will be blinded before, during or after the trial.

### Treatment

#### Control

Participants in the control group receive no medical rehabilitation and are identified through propensity score matching.

#### Intervention

Participants in the treatment group will take part in a medical rehabilitation programme based on current treatment standards and guidelines for the rehabilitation of back pain [34]. The medical rehabilitation programmes are multimodal and consist of three to 4 h of therapy per day, including sports and exercise therapy, physiotherapy, occupational therapy, massage and other physical therapies, social and psychological counselling, patient education, pain management and relaxation training. They focus on the functional limitations of the musculoskeletal system and aim to restore physical abilities in order to promote participation in work and daily life. The duration of the rehabilitation programme is determined initially by the GPI North and GPI Central Germany and is usually about 3 weeks. Rehabilitation services may be provided as inpatient or outpatient programmes. Patients with musculoskeletal diseases spend 23 days at the rehabilitation centre on average [44], with sports exercise being the most frequent therapy during the 3-week rehabilitation (>13 h per week, 74% of all therapies) [44]. The rehabilitation centre and the patient may arrange to extend the programme. The patient may stop the rehabilitation programme ahead of schedule on request.

Applications to use rehabilitation services, the related decisions, and the utilisation of these services are documented in the GPI registers and data relating to the first year of the study will be extracted from these records.

### Participants

Employed persons aged 45 to 59 years will be included. The exclusion criteria are having applied for or used medical rehabilitation services during the last 4 years and previous requests for or receipt of disability pension benefits. Samples will be drawn in equal proportions from the populations of the GPI North (*n* = 22,500) and GPI Central Germany (*n* = 22,500). The samples will be stratified by sex in a ratio of 1:1 and by sickness absence benefits in a ratio of 2:1 (less than 1 week of sickness absence benefits vs. at least 1 week of sickness absence benefits). In the first quarter of the first year, a baseline survey will be performed to assess the 3-month prevalence of back pain (i.e., back pain at any time during the past 3 months including today). The localisation of back pain will be represented on a pain drawing [1]. In accordance with previous large, epidemiological studies in Germany, back pain is defined as pain anywhere in the area between C7 and the gluteal folds [45–47]. Only persons who report some kind of back pain in the baseline survey will be followed in our prospective analysis.

### Sample size estimation

**Fig. 1:**
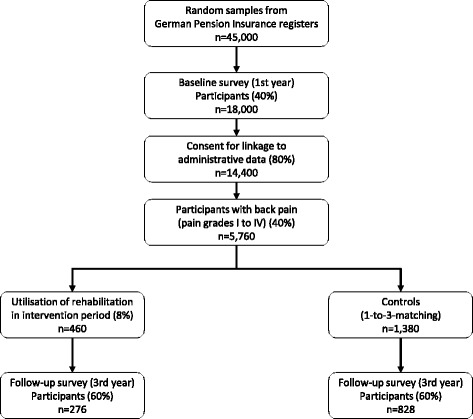
Flow of participants

Estimates for baseline response (40%), consent to linking survey with administrative data (80%), back pain (40%) [48], utilisation of rehabilitation (8%) and response to follow-up (60%) are derived from other studies. In case of a 1-to-3-matching of rehabilitants and controls, the final sample of 276 rehabilitants and 828 untreated controls is sufficient to detect a minor difference in the primary outcome between rehabilitants and controls (standardised mean difference = 0.2; two-tailed alpha = 0.05) with a power of 0.82. (Fig. 1).

### Recruitment

After the sample has been drawn potential study participants will be contacted by post by the GPI. The first mailing of the baseline survey will include a personalised cover letter, information about the study, a consent form, and two envelopes (a prepaid envelope and a small envelope for the consent form). The prepaid envelope is to be used to send the completed questionnaire and the envelope containing the consent form to the university. The project staff at the university will collect the consent form envelopes and pass them on to the relevant GPI employees without opening them, as they contain personal data. The GPI employees will check off those who have provided informed consent against a study list and inform the scientists at the university by transferring the identification numbers for consenting participants. Participation is voluntary and participants are free to withdraw their consent at any time without any consequences.

One, four and five weeks after the initial mailing of the questionnaire reminders will be sent to potential study participants. The first reminder takes the form of a short thank-you letter to remind the recipient about the study and the questionnaire they received recently. The second reminder is sent 3 weeks after the first reminder and contains all of the above-mentioned study material. This reminder is only sent to persons who have not yet returned their questionnaire. The third and final reminder is sent 1 week after the second reminder, once again it highlights the importance of the recipient’s participation in the study and thanks them in advance for their support. The final reminder also contains a link to the study website (www.rehab-bp.de) which will inform about the study and its findings.

During the third study year, a second survey will be conducted. The format is the same as for the first survey: a first mailing comprising a cover letter, questionnaire and prepaid envelope, a second mailing consisting of a short reminder, a third mailing containing the same material as the first, sent only to those who have not yet returned their questionnaires, and a fourth mailing consisting of a final short reminder. The schedule for enrolment, interventions and assessments is displayed in Table 1.

**Table 1:**
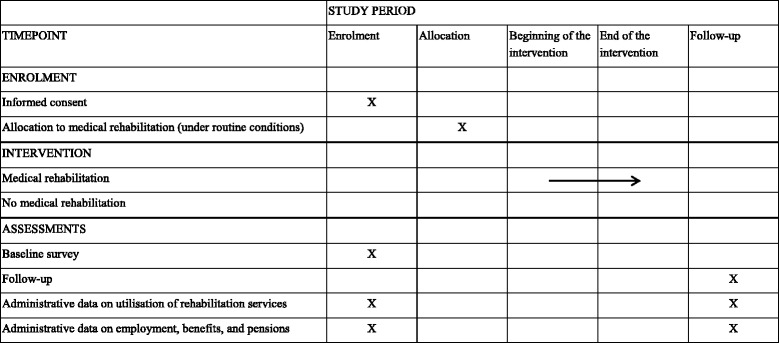
Time schedule of enrolment, interventions and assessments

### Data management

Questionnaires will be scanned and verified by an electronic data capture system and exported to statistical software packages for further analysis. Scanning and verifying will be done by trained research assistants who will compare the imported data with the original questionnaire data. Administrative data will be extracted by the GPI North and GPI Central Germany and personal data will be removed and replaced by the unique study identifier. These administrative data will be transferred to the principal investigators (MB, WM). Questionnaire and administrative data can be linked via the unique study identifier.

The authors of the protocol will be responsible for data management in the study. Data access will be limited to the authors and the team of research assistants.

### Outcomes and other measures

The questionnaire used in this study will include items on various barriers to rehabilitation utilisation, primary and secondary outcomes and some additional variables. Data on additional outcomes will be extracted from administrative records (i.e. individual pension insurance accounts). These administrative data will be provided by the GPI North and GPI Central Germany. A complete list of all measured constructs, measurement points and expected scaling is shown in Table 2.

**Table 2:** Measures, assessment, expected scaling and measurement points

Outcome	Source and reference	Total score	Scaling	Baseline	Follow-up
Primary Outcome					
Pain disability	CPQ [48, 49]	0 to 100	metric	X	X
Secondary Outcomes					
Pain intensity	CPQ [48, 49]	0 to 100	metric	X	X
Days of disability	CPQ [48, 49]	0 to 90	metric	X	X
Pain self-efficacy	PSEQ [50, 51]	10 to 60	metric	X	X
Fear avoidance beliefs	Modified FABQ subscales [52, 53]	0 to 18	metric	X	X
General health	COPSOQ [54, 55]	0 to 10	metric	X	X
Depressive symptoms	PHQ-8 [56, 57]	0 to 24	metric	X	X
Work ability	WAS [58]	0 to 100	metric	X	X
Subjective prognosis of employability	SPE [59]	0 to 3	ordinal	X	X
Healthcare utilisation:	Own development				
Use of pharmaceuticals			binary	X	X
Outpatient visits to physicians			metric	X	X
Utilisation of therapy			metric	X	X
Hospital stay			binary	X	X
Days of sick leave		0 to 365	metric	X	X
Days in receipt of sickness absence benefits	GPI registers		metric	X	X
Days in regular employment	GPI registers		metric	X	X
Application for disability pension	GPI registers		binary		X
Grant of a disability pension	GPI registers		binary		X
Other measures					
Comorbidity	Modified subscale of the SCQ [60, 61]	0 to 15	metric	X	
Health behaviour	Own development				
Physical exercise			binary	X	
Cigarette smoking			binary	X	
Body Mass Index			metric	X	
Self-help group or organisation			binary	X	
Employment status	Own development		nominal	X	
Job profession	Own development		nominal	X	
Working hours	Own development		ordinal	X	
Fixed-term job contracts	Own development		nominal	X	
Shift work	COPSOQ [54, 55]		nominal	X	
Size of enterprise	COPSOQ [54, 55]		ordinal	X	
Physical demands	FEBA [62]	0 to 15	metric	X	
Psychological demands	COPSOQ [54, 55]	0 to 100	metric	X	
Support by supervisor and colleagues	COPSOQ [54, 55]	0 to 100	metric	X	
Atmosphere at work	COPSOQ [54, 55]	0 to 100	metric	X	
Job insecurity	COPSOQ [54, 55]	0 to 100	metric	X	
Overall job satisfaction	COPSOQ [54, 55]	0 to 100	metric	X	
Workplace bullying	[63]	0 to 100	metric	X	
Cognitions about experiences with rehabilitation services	[64]				
Former medical rehabilitation services			binary	X	
Subjective need for rehabilitation			binary	X	
Intention to apply for medical rehabilitation			binary	X	
Knowledge of rehabilitation application procedures			metric	X	
Negative outcome expectations			metric	X	
Social support from family and friends			metric	X	
Social support from physicians and therapists			metric	X	
Socio-demographic data	Own development		metric/nominal	X	

*COPSOQ* Copenhagen Psychosocial Questionnaire, *CPQ* Chronic Pain Grade Questionnaire, *FABQ* Fear-Avoidance Beliefs Questionnaire, *GPI* German Pension Insurance, *FEBA* Fragebogen zur subjektiven Einschätzung der Belastungen am Arbeitsplatz (questionnaire on job demands), *PSEQ* Pain Self-Efficacy Questionnaire, *PHQ-8* Patient Health Questionnaire (8-item version), *SCQ* Self-Administered Comorbidity Questionnaire, *SPE* subjective prognosis of employment status, *WAS* work ability score

#### Primary outcome

Pain disability will be the primary outcome in our analyses of the effectiveness of medical rehabilitation and will be assessed at baseline and follow-up. Three items from the German version of the Chronic Pain Grade Questionnaire will be used to assess pain disability [48, 49]. These items deal with the extent to which pain interferes with important life domains, including daily living, leisure time and work. Because it includes the work domain this measure also covers work ability, which is the primary focus of GPI-approved rehabilitation services. The German version of the Chronic Pain Grade Questionnaire has been shown to be a reliable, valid and useful instrument [49]. Items are scored from zero to ten points, with higher scores indicating greater disability. Item scores are averaged and multiplied by ten to give a total pain disability score ranging from 0 to 100 points.

#### Secondary outcomes

The secondary outcomes of our analyses of the effectiveness of rehabilitation will be assessed at baseline and follow-up.

##### Pain intensity and disability days

Four items from the Chronic Pain Grade Scale will be used to assess pain intensity (three items) and days of disability (one item) [48, 49]. The pain intensity items cover current, worst and average pain in the last 3 months. The items are scored from zero to ten points, with higher values indicating stronger pain. Items are averaged and multiplied by ten to give a total pain intensity score of between 0 and 100 points. Days of disability are the number of days in the previous 3 months when the respondent could not carry out his or her usual activities due to pain. The variables pain disability, pain intensity and days of disability are used to derive a pain grade. Pain grades range from no pain to severely limiting pain [2, 48, 49]. Pain grade I represents low intensity pain associated with limited disability, grade II represents high intensity pain and limited disability, grade III represents moderate disabling pain regardless of pain intensity, and grade IV represents severely disabling pain regardless of pain intensity [48].

##### Pain self-efficacy

Self-efficacy with respect to coping with back pain will be assessed with the German adaptation of the Pain Self-efficacy Questionnaire (PSEQ; German: Fragebogen zur Erfassung der schmerzspezifischen Selbstwirksamkeit, FESS) [50, 51]. The FESS measures belief that one will be able to perform various activities despite one’s pain. The ten items are rated on a 6-point scale from 1 (‘not at all confident’) to 6 (‘completely confident’). Total scores range from 10 to 60, with higher scores indicating greater pain self-efficacy. The FESS has been shown to be a valid instrument for the evaluation of therapeutic success in the context of pain research [51].

##### Fear avoidance beliefs

The German version of the Fear Avoidance Belief Questionnaire (FABQ) [52, 53] comprises two subscales. In this study three items of the physical activity scale will be used to assess belief that physical activity causes or compounds back pain. Additionally, three items of the work subscale will be used to assess beliefs about how work might affect one’s back pain. The 7-point scale ranges from 0 (‘completely disagree’) to 6 (‘completely agree’). Score for items from both scales will be added to yield a sum score ranging from 0 to 18 points, with higher values indicating stronger fear avoidance beliefs.

##### General health

General health will be assessed with one item from the Copenhagen Psychosocial Questionnaire with an 11-point scale (0 ‘worst imaginable health state’ to 10 ‘best imaginable health state’) [54, 55].

##### Depressive symptoms

Depressive symptoms will be measured using the 8-item depression module of the Patient Health Questionnaire (PHQ-8), which is recommended for research purposes [56, 57]. Items assess the frequency of depressive symptoms during the last 2 weeks and responses are given on a 4-point scale (0: ‘not at all’ to 3: ‘nearly every day’). Total scores range from 0 to 24 points.

##### Work ability

Self-rated work ability is assessed using the Work Ability Score (WAS) [58]. This 11-point scale ranges from 0 (‘completely unable to work’) to 10 (‘maximal work ability’).

##### Subjective prognosis of employability

Three items will be used to assess whether participants believe they will remain at work until retirement, whether they assume that their health is permanently jeopardised and whether they intend to request a disability pension [59]. Dichotomous item scores are summed to give a total score ranging from 0 to 3 points, with higher values indicating a less favourable prognosis.

##### Healthcare utilisation

Single items will be used to assess use of three types of pharmaceuticals over the last 3 months: pain-reducing medication, mood-enhancing medication, medication for other health complaints. Response categories are ‘no’ (0 points), ‘regularly’ (e.g. daily) (1 point) and ‘as needed’ (1 point). Outpatient visits to physicians will be assessed as the number of visits in the last 12 months. The utilisation of outpatient therapies (e.g. physiotherapy) will be assessed as the number of therapies units used over the last 12 months. Hospitalisation within the last 12 months will be captured as a dichotomous variable (0: ‘no’ and 1: ‘yes’). The number of days of sick leave during the last 12 months will also be assessed.

##### Sickness absence benefits and disability pensions

Data on days in receipt of sickness absence benefits, days in regular employment, applications for and granting of a disability pension will be extracted from the GPI registers during the third year of the study. These variables are the secondary outcomes in our analyses of the effectiveness of medical rehabilitation.

#### Other measures

Data on other variables will be collected at baseline in order to provide a description of the study sample, to determine potential barriers to the use of rehabilitation services, to gain variables for estimating the propensity score, and to identify variables that might influence the effect of rehabilitation services.

##### Comorbidity

Comorbidity will be assessed using an adapted version of the German version of the Self-Administered Comorbidity Questionnaire [60, 61], using only the ‘impairments because of the corresponding health problem’ subscale.

##### Health behaviour

Physical exercise, cigarette smoking, body mass index and membership of a self-help group or organisation will be assessed as indicators of health-related behaviour.

##### Work stress and work environment

Physical job demands will be measured using five 4-point items that yield a total score ranging from 0 to 15 points [62]. Psychological job demands (six items), job insecurity (two items), support by supervisor and colleagues (two items), atmosphere at work (one item), and overall job satisfaction (one item) will be assessed using the short version of the Copenhagen Psychosocial Questionnaire [54, 55]. Total scores for these variables range from 0 to 100 points. Workplace bullying will be assessed with a single 5-point item [63]. We will also capture information about professional title, weekly working hours, fixed-term job contracts, size of enterprise and shift work.

##### Cognitions about and experiences with rehabilitation services

Motivational factors related to application for rehabilitation services will be assessed using a set of questions that was recently developed for the Third German Sociomedical Panel of Employees [64]. The questions include items about subjective need for rehabilitation (and reasons for not having such a need), intention to apply for a medical rehabilitation programme (and reasons for not intending to apply), knowledge of rehabilitation application procedures, negative outcome expectations, and support for the application for rehabilitation from family and friends and physicians and therapists.

##### Socio-demographic data

Age and sex will be derived from the GPI registers. Data on net income, job position, and educational level will be self-reported. Assessment of migrant status will be assessed based on Schenk’s recommendations [65]. Partnership status (single or partnered) and size of household, including number of children under 14 years and number of family members with a special need for care will also be assessed. Stress associated with housework and family commitments will be rated using two 11-point items (0: ‘not at all stressed’ to 10: ‘very stressed’) [66].

### Statistical analysis

#### Analysing barriers to rehabilitation utilisation

To identify barriers to the utilisation of medical rehabilitation services we will compare persons with back pain who complete a medical rehabilitation with persons with back pain who do not. We will identify barriers to use of rehabilitation services across the entire course of the rehabilitation process, from recognition of need for rehabilitation and applying for a medical rehabilitation programme, to applying successfully for a grant, participating in a rehabilitation programme and completing the programme. Different barriers apply to each step and these will be identified by analysing group differences in baseline values of the outcome and control variables at each stage of the process. The first step in the analysis will be univariate analyses: *t* tests and analyses of variances for continuous variables, Mann–Whitney *U* tests for ordinal variables and chi-squared tests for binary variables. The second step will consist of logistic regression analyses to identify the most important predictors of rehabilitation intention, rehabilitation application, acceptance onto a rehabilitation programme and use of rehabilitation. In the third step we will estimate generalised structural equation models to describe the direct and indirect effects of potential barriers.

#### Propensity score matching

Rehabilitation participants and untreated subjects will be matched by propensity scores to achieve a balanced sample and enable calculation of unbiased estimates of effectiveness [67–72]. The propensity score is the conditional probability of receiving the treatment under evaluation (i.e. the medical rehabilitation programme) given the vector of observed background variables. Matching by propensity scores allows one to obtain a sample in which the treated and untreated groups are balanced, if there is sufficient overlap between their propensity scores. Propensity score matching also allows one to avoid the dimensionality problem that affects conventional direct matching procedures involving multiple variables, e.g. age, sex, socioeconomic state, depression, work ability, by reducing the matching requirement to a single dimension.

The propensity score will be estimated using a logistic regression model including all variables that are associated with treatment allocation. For every treatment case three persons with a similar propensity score will be selected from the larger pool of non-treatment cases. Re-sampling with replacement will be used to reduce bias. If it is necessary to achieve a balanced sample, a caliper of one-quarter of the propensity score will be used during re-sampling. Additional matching schemes will be used in the sensitivity analysis.

Standardised percentage bias will be calculated as an indicator of bias before and after matching due to differences in observed sample characteristics. This is the difference between sample means in the intervention and control groups relative to the square root of the average of the sample variances in both groups [73]. Multiple imputations will be used to fill in missing data before propensity scores are estimated. The propensity scores for each record will be averaged across the completed datasets and propensity score matching will be performed with these averaged scores [74].

#### Estimating the treatment effect

The average treatment effect for the treated cases will be estimated with weighted linear regression models in the case of continuous outcomes and generalised linear models in the case of binary outcomes (see above: primary outcome and secondary outcomes) [75]. Robust standard errors will be calculated.

#### Moderator analyses

Moderator analyses will be performed to clarify whether individual characteristics modify the treatment effect. The moderating effects of individual characteristics will be modelled in terms of multiplicative interaction terms for the treatment indicator and potential moderators. In the case of continuous moderators, these variables will be *z*-standardised [76]. Moderators will be tested in order to identify the patients who would benefit most from medical rehabilitation.

## Discussion

Our cohort study examines barriers patients with CBP face in attempting to access rehabilitation; it addresses the lack of prospective, longitudinal studies in this area of research. Barriers to accessing and using rehabilitation services are analysed at several time points. Furthermore this study appraises the effectiveness of routine German medical rehabilitation services for patients with back pain. The findings of this study will be published in peer-reviewed journal articles and presented at conferences.

## Trial status

Recruitment has started and is ongoing (Additional file 1).

## Additional files

Additional file 1: Reports the trial registration data. (DOCX 16 kb)

Additional file 2 Is the letter telling potential participants about the study’s objectives. (DOCX 15 kb)

## Abbreviations

CBP: Chronic back pain
COPSOQ: Copenhagen Psychosocial Questionnaire
CPG: Chronic Pain Grade Questionnaire
FABQ: Fear-Avoidance Beliefs Questionnaire
FEBA: Fragebogen zur subjektiven Einschätzung der Belastungen am Arbeitsplatz
GPI: German Pension Insurance
PHQ-8: Patient Health Questionnaire (8-item version)
PSEQ: Pain Self-Efficacy Questionnaire
SCQ: Self-Administered Comorbidity Questionnaire
SPE: Subjective prognosis of employment status
WAS: Work ability score
