# Supplementary material for: Rehabilitation access and effectiveness for persons with back pain: the protocol of a cohort study (REHAB-BP, DRKS00011554)
Source: BMC Public Health. 2017 Jul 14;18:22. doi: 10.1186/s12889-017-4588-x (PMC5512943; doi:10.1186/s12889-017-4588-x)
Supplement: Supplementary file 1 — Reports the trial registration data. (DOCX 16 kb) [file 12889_2017_4588_MOESM1_ESM.docx]

## Additional file 1: Trial Registration Data

| Data category | Information |
| --- | --- |
| Primary registry and trial identifying number | German Clinical Trials Register DRKS00011554 |
| Date of registration in primary registry | January 26, 2017 |
| Secondary identifying numbers | U1111-1162-7501 |
| Source(s) of monetary or material support | German Research Foundation |
| Primary sponsor | Matthias Bethge: matthias.bethge@uksh.de  Wilfried Mau: wilfried.mau@medizin.uni-halle.de |
| Secondary sponsor(s) | - |
| Contact for public queries | Matthias Bethge: matthias.bethge@uksh.de  Wilfried Mau: wilfried.mau@medizin.uni-halle.de |
| Contact for scientific queries | Matthias Bethge, University of Lübeck, Institute of Social Medicine and Epidemiology, Ratzeburger Allee 160, 23562 Lübeck, Germany, Telephone: +49 451-50051280, Email: matthias.bethge@uksh.de |
|  | Wilfried Mau, Martin Luther University Halle-Wittenberg, Institute of Rehabilitation Medicine, Magdeburger Str. 8, 06112 Halle, Germany Telephone: +49 345-5574206, Email: wilfried.mau@medizin.uni-halle.de |
| Public title | Rehabilitation access and effectiveness cohort study for persons with back pain |
| Scientific title | Rehabilitation access and effectiveness cohort study for persons with back pain - REHAB-BP |
| Countries of recruitment | Germany |
| Health condition(s) or problem(s) studied | M40-M54 - Dorsopathies |
| Intervention(s) | Arm 1: Medical rehabilitation services (with funding by the German Pension Insurance) |
|  | Arm 2: No medical rehabilitation |
| Key inclusion and exclusion criteria | Inclusion criteria: Employed persons, minimum age: 45 years, maximum age: 59 years, sex: male or female |
|  | Exclusion criteria: Persons who requested or utilised medical rehabilitation services during the past four years and persons who have requested or received a disability pension. |
| Study type | Non-intervention |
|  | Allocation: non-randomised controlled trial |
|  | Masking: open (masking not used); Control: Control group receives no treatment; Assignment: parallel |
|  | Study design purpose: treatment |
|  | Phase: IV |
| Date of first enrolment | March 20, 2017 |
| Target sample size | 45000 |
| Recruitment status | Pending |
| Primary outcome(s) | Pain disability: 3 items assess impairments affecting everyday life, leisure time and work; scores range from 0 to 100. Higher values indicate greater impairment. The primary outcome will be assessed by questionnaire at baseline and follow-up (German version of the Chronic Pain Grade (Klasen et al. 2004)). |
| Key secondary outcomes | The following secondary outcomes will be assessed by questionnaire at baseline and follow-up:  - pain intensity (0 to 100 points) and days of disability: German version of the Chronic Pain Grade (Klasen et al. 2004)  - pain self-efficacy (10 to 60 points): German adaption of the Pain Self-efficacy Questionnaire (Mangels et al. 2009)  - fear avoidance beliefs: adapted version of two subscales (physical activity, 0 to 18 points; work as a cause of pain, 0 to 18 points) of the German version of the Fear Avoidance Belief Questionnaire (FABQ) (Pfingsten et al. 1997)  - general health (0 to 10 points): single item (Kristensen et al. 2010)  - depressive symptoms (0 to 24 points): 8-item depression module of the Patient Health Questionnaire (PHQ-8) (Gräfe et al. 2004)  - self-rated work ability (0 to 10 points): Work Ability Score (WAS) (Ilmarinen 2007)  - subjective prognosis of employment status (0 to 3 points): 3-item scale (Mittag and Raspe 2003)  - healthcare utilisation: use of pharmaceuticals, visits to physicians, outpatient therapy e.g. physiotherapy, hospitalisation, and days of sick leave  The following secondary outcomes will be extracted from the pension insurance accounts for 2016 and 2018:  - days of sickness absence benefits  - days in regular employment  - disability pensions (application, rejection and granting)  To enable estimation of propensity scores some additional measures will be assessed at baseline:  - comorbidity (0 to 15 points): adapted version of the German version of the Self-Administered Comorbidity Questionnaire (SCQ-D) (Streibelt et al. 2012)  - health behaviour: physical exercise, smoking, body mass index and membership of a self-help group  - socio-demographic data: migration, educational level, job position, net income, partnership status, size of household, number of children, number of family members with a special need for care, stress due to housework and family commitments (2 items, values ranging from 0 to 10) (Worringen and Benecke 2001)  - work environment: employment status, profession, fixed-term job contracts, shift work, size of enterprise  - physical demands (FEBA) (0 to 15 points) (Slesina 1987)  - measures of the Copenhagen Psychosocial Questionnaire (COPSOQ) (0 to 100 points): psychological demands, support from supervisor and colleagues, atmosphere at work, job insecurity, overall job satisfaction (Nuebling and Hasselhorn 2010)  - workplace bullying (Institut für Arbeitsmarkt- und Berufsforschung 1999)  To assess cognitions about and experiences with rehabilitation services, additional variables will be assessed at baseline and follow-up (Spanier et al. 2016):  - previous use of medical rehabilitation services  - subjective need for rehabilitation  - intention to apply for access to medical rehabilitation  - knowledge of rehabilitation application procedures  - negative outcome expectations  - social support from family/friends and physicians/therapists |
