# Supplementary material for: Rehabilitation access and effectiveness for persons with back pain: the protocol of a cohort study (REHAB-BP, DRKS00011554)
Source: BMC Public Health. 2017 Jul 14;18:22. doi: 10.1186/s12889-017-4588-x (PMC5512943; doi:10.1186/s12889-017-4588-x)
Supplement: Supplementary file 2 — Is the letter telling potential participants about the study’s objectives. (DOCX 15 kb) [file 12889_2017_4588_MOESM2_ESM.docx]

## Additional file 2: Participant information letter

Dear …

As you know, German Pension Insurance is an important partner in the social security system. The German Pension Insurance is responsible not only for administering pension insurance contributions and pension payments, but also for rehabilitation services. The German Pension Insurance funds these services so that you can continue working as long as possible.

Today we would like to ask you kindly to help us by participating in our survey.

*Who is conducting this research project?*

This research project is being carried out by the University of Lübeck and the Martin-Luther-University Halle-Wittenberg and it is funded by the German Research Foundation. The leaders of this research project are Prof. Matthias Bethge (University of Lübeck, Institute for Social Medicine and Epidemiology, Section Rehabilitation and Work, Ratzeburger Allee 160, Lübeck) and Prof. Wilfried Mau (Martin Luther University Halle-Wittenberg, Medical Faculty, Institute of Rehabilitation Medicine, Magdeburger Straße 8, Halle).

*What is this project about?*

Chronic conditions have a big influence on one’s quality of life and ability to work and are one of the main factors responsible for early retirements. Medical rehabilitation services can offer one an opportunity to improve one’s health and quality of life and prevent early retirement due to impaired ability to work. However, studies show that despite suffering impairments only a few people request access to rehabilitation services and they tend to wait quite a long time before doing so. Because of this the researchers want to identify the factors that make it easier or more difficult to apply for medical rehabilitation services. They also want to verify the effectiveness of medical rehabilitation services.

Your participation would be entirely voluntary. Before you have to decide whether or not you want to participate we will explain the project itself and how we will handle your personal data in more detail. You are also welcome to have a look at the attached questionnaire.

*How does it work? How can you help us?*

If you decide to participate in this survey then please sign the declaration of consent and fill out the questionnaire. The researchers want to use two sources of data. First, the information from the questionnaire and second, some information selected from your insurance account (such as information about requests you have made for rehabilitation services and disability pension and the outcomes of such requests, diagnoses, the type of rehabilitation involved and number of days of employment, unemployment and sickness absence benefits). Please send us your completed questionnaire completely in the prepaid envelope, together with the signed consent form, which you should seal in the second envelope. In two years’ time we will send you another questionnaire.

*Data protection: How do we handle the collected data?*

To assure that your personal data is protected, we will assign a research number to your name and this will be saved in a study list. This list is necessary for the distribution of the questionnaires and will be managed by the German Pension Insurance. The list will not be accessible to the researchers. Once all the data have been collected the list will be destroyed.

Your declaration of consent will be forwarded to the German Pension Insurance in its sealed envelope and will not be opened by the researchers. This procedure ensures that neither the researchers nor German Pension Insurance can relate the information from your questionnaire to you as a person. All the analyses will be carried out on pseudonymised data, which means that your data will only be identified by your research number, not your name or other personal information.

*Independence of the researchers*

The project is supported by the German Pension Insurance, who will act as a point of contact between you and the researchers. The insurance company will send out the letters with the questionnaires, and will select, in strict accordance with data protection regulations, certain important pieces of information from your pension account. This information will be forwarded to the researchers in pseudonymised form. The research team is responsible for the conduct of the research project, for the activities involved and for ensuring that all the research procedures comply with the data protection regulations. Your decision about participating in the project will not have any influence on the services you receive from the German Pension Insurance. Only the research team will have access to the information you provide in the questionnaire.

*Participation is voluntary*

Your participation in our survey is voluntary. Your data will only be used if you sign the declaration of consent and send it back to us with a completed questionnaire. If you don’t want to participate, you don’t have to do anything. If we contact you in the next few weeks, you don’t have to respond. There will not be any negative consequences for you if you decide not to participate in the survey.

You can decide to end your involvement in the study at any time, even after you have signed the declaration of consent and you do not have to give a reason for doing so. If you no longer want to be involved in the study please inform your personal contact at the German Pension Insurance. If you do this your details will be deleted from the study list, no further data will be collected from your insurance account and you will not be sent any more questionnaires. The data that has already been collected will be used anonymously.

*Invitation to participate*

The success of the research study depends on recruiting a high number of participants, so we kindly ask you to participate. If you want to support the project by taking part please put your completed questionnaire and consent form (in its sealed envelope) in the prepaid envelope. We will pay the postage costs.

If you have any further questions please contact (personalised contact person).

Thank you for your participation and support.

Yours sincerely
